# What Is Next for NTDs in the Era of the Sustainable Development Goals?

**DOI:** 10.1371/journal.pntd.0004719

**Published:** 2016-07-07

**Authors:** James Smith, Emma Michelle Taylor

**Affiliations:** 1 Centre of African Studies, University of Edinburgh, Edinburgh, United Kingdom; 2 Department of Geography, Environmental Management and Energy Studies, University of Johannesburg, Johannesburg, South Africa; Task Force for Global Health, UNITED STATES

## Introduction

In a previous article, we outlined the efforts of the Neglected Tropical Diseases (NTD) lobby to overcome the diseases' snub in the 2000 Millennium Development Goals (MDGs) and to campaign for inclusion in the post-2015 development agenda [1]. In doing so, we noted the extraordinary achievements made by the lobby despite its view from outside of the MDG juggernaut, which singled out just three diseases for special consideration (HIV/AIDS, Malaria, and, latterly, Tuberculosis), leaving a group of previously disparate diseases to be formulated within a plan for inclusion. The plan resulted in a three-pronged attack forged on advocacy, policy, and science.

The NTD lobby has grown significantly since the original 2003 WHO/Deutsche Gesellschaft für Technische Zusammenarbeit (GTZ) meetings were convened to float the idea that the NTDs should be reconceptualised and tackled as a group moving forward. The original core group—Peter J. Hotez, David Molyneux, and Alan Fenwick (along with WHO’s Lorenzo Savioli and others)—has since been joined by representation from academia (e.g., *PLOS Neglected Tropical Diseases*), the third sector (e.g., the Global Network, the Neglected Tropical Disease Non-Governmental Development Organization Network), the pharmaceutical industry, donor agencies, and the philanthropic sector (cf. Uniting to Combat NTDs). The lobby is also formally supported by the World Health Organisation (through the WHO Roadmap [2] and WHA Resolution 66.12 [3]) and by the disease endemic country governments that have signed the Addis Ababa NTD Commitment. Yet, despite boasting swelling ranks and a number of sizeable achievements in the intervening period—for instance the landmark London Declaration—the NTD lobby has remained interested in the more mainstream development agenda. The advocacy goal has always been the formal inclusion of NTDs in the successor framework to the Millennium Development Goals: the post-2015 Sustainable Development Agenda.

On 1 January 2016, the Sustainable Development Goals (SDGs or *Global Goals*) came into force. The particulars of the goals were finalised at the United Nations summit in September 2015, and, as hoped, NTDs gained a special mention in goal 3.3: "By 2030, end the epidemics of AIDS, tuberculosis, malaria, and neglected tropical diseases and combat hepatitis, waterborne diseases, and other communicable diseases" (p 16) [4]. But what is this mention worth and what are its implications? The SDGs are similar to the MDGs in that they represent time-bound targets and retain a focus on ending world poverty, but they break with the original eight MDGs in terms of their colossal scope. Composed of 17 goals and 169 targets, the SDGs are the result of an extensive three-year consultation involving multiple perspectives from government, civil society, expert groups, the private sector, and individuals. The MDGs were regularly critiqued as restricted in focus and top-down, something which cannot be levelled against the SDGs. But might the warning of the High-Level Panel that originally drafted the framework document have been ignored—"The international community will need to ensure that a single, sustainable framework agenda is not overloaded with too many priorities. A product of compromise rather than decisions…"? (p 14) [5].

The post-2015 vision—as articulated through the SDGs—is to create a single universal agenda in which the social, economic, and environmental dimensions of sustainability are integrated. Whereas the MDGs were criticised for targeting the low-hanging fruit, the post-2015 agenda is underpinned by the tenet “leave no one behind.” As the UN summit drew closer in 2015, we noted how this principle, coupled with the post-2015 focus on environmental issues, provided a number of advocacy issues behind which the NTD lobby began to position itself: equity, poverty, water sanitation and hygiene (WASH), climate change, quality education, NTD-related disability, and nutrition. In this paper, we reflect on the NTD lobby’s efforts to gain traction in the wide-ranging post-2015 agenda by making the case that NTDs are crosscutting and by lobbying for NTD indicators.

## Framing the NTDs as Crosscutting and Underpinning

SDG target 3.3 secured a place for NTDs in the post-2015 Sustainable Development Agenda. Yet, the significance of this nod is slightly downplayed by the exhaustive list of health concerns on either side of it, as detailed in the overarching Goal 3, “Ensure healthy lives and promote well-being for all at all ages,” which is accompanied by 13 targets covering everything from maternal health to non-communicable disease and traffic accidents. Perhaps anticipating the white noise of being part of such a large agenda, the NTD lobby has been ahead of the curve in formulating a case that NTDs should be anchored across the broader agenda, whether that be rhetorically (the case that NTDs are both outcomes and drivers of poverty has already been made [6]) by partnering directly with other lobbies such as WASH to support multidisciplinary interventions or literally by proxy by suggesting that NTDs should be used as tracer indicators for other SDG targets.

Of course, framing the MDGs as crosscutting is not a new strategy for the NTD lobby. In a series of policy papers, which launched the campaign to see NTDs conceptualised and tackled as a discrete group, NTDs were portrayed as direct hindrances to the attainment of the MDGs; conversely, tackling them head on was portrayed as directly beneficial to seven out of the eight goals [7]. But while that advocacy was often left vague, the current campaign is striving to make explicit the connections between NTDs, the broader goals, and, in particular, the value of using NTDs as tracers for a number of the SDG targets to monitor equity in the new post-2015 agenda. The WHO’s Director for the Department of NTDs Dirk Engels has argued that this is necessary “precisely because NTD endemic populations are the least likely to have access to these services [like Universal Health Coverage (UHC) and access to safe water] at present” (p 224) [8].

The WHO’s *Third Report on NTDs* provides a concrete example of how this approach is being realised [9]. Here, WHO is unequivocal that control of NTDs would not only have positive knock-on effects on many of the proposed SDG targets but could also, in certain instances, be taken as proxy indicators for measuring progress toward them. Accordingly, NTD control measures are put forward as a test case for UHC (SDG 3.8). In this manner, the burden of NTDs is suggested as “a proxy for inequitable access to the systems—especially health systems—through which people improve their health and wealth” (p 12) [9], and UHC is depicted as “one of the most powerful social equalizers among all policy options” (Margaret Chan cited in [p x] [9]). WHO’s Dirk Engels has since spoken about “mainstreaming” the NTD’s medical interventions (preventative chemotherapy and intensive disease management) in the new agenda by moving them into national systems and thus reinforcing the case that the NTDs can be used as a tracer indicator for UHC (Engels addressing the Royal Society of Tropical Medicine and Hygiene’s [RSTMH] Elimination Event 25 September 2015) alongside other tracer interventions such as child immunisation and antiretroviral therapy.

Climate change (SDG 13.3) is another area where the WHO report suggests NTDs control could serve as a proxy indicator. The argument goes that because several of the NTDs (e.g., Dengue) involve an insect or animal vector, they are highly sensitive to climate variation; therefore, “Changes in incidence can serve as an early and measurable signal of the health effects of climate change” (p ix) [9].

While the report doesn’t go so far as to suggest that NTDs be used as a proxy for extreme poverty, the pro-poor nature of the initiative to combat NTDs is emphasised—“with such huge numbers affected, controlling these diseases paves the way for an exodus from poverty” (p ix) [9]. This builds on a body of work that has sought to establish NTD interventions for the seven most prevalent diseases as a “best buy” in global public health [10,11]. Positive knock-on effects are argued for other SDG targets in the third WHO report as well, specifically nutrition, water, and sanitation.

In August 2015, the WHO announced a *Joint Global Strategy* for the NTD and WASH sectors [12], finally seeking to tap into the natural synergies between the two camps. In formalising this collaboration, the WHO has come up with another “bright idea” for the SDG framework (Dirk Engels addressing the RSTMH Elimination Event 25 September 2016): use NTDs as a tracer for equitable access to WASH, focusing on SDGs 6.1 (drinking water) and 6.2 (sanitation) (ibid and [8]). In the final run up to the SDG announcement, Hotez and Herricks [13] mirrored WHO’s approach by suggesting a way in which helminth infection could theoretically be used as a proxy indicator for human development. Tables 1 and 2 summarise the SDG goals and targets that have been argued to boast direct or indirect associations to NTDs.

**Table 1:** Sustainable Development Goals with direct and indirect associations to NTDs.

Sustainable Development Goals
Goal 1. End poverty in all its forms everywhere.
Goal 2. End hunger, achieve food security and improved nutrition, and promote sustainable agriculture.
Goal 3. Ensure healthy lives and promote well-being for all at all ages.
Goal 4. Ensure inclusive and equitable quality education and promote lifelong learning opportunities for all.
Goal 6. Ensure availability and sustainable management of water and sanitation for all.
Goal 13. Take urgent action to combat climate change and its impacts.
Goal 17. Strengthen the means of implementation and revitalise the Global Partnership for Sustainable Development.

**Table 2:** SDG targets with direct and indirect associations to NTDs.

SDG Targets
Target 3.3. By 2030, end the epidemics of AIDS, tuberculosis, malaria, and neglected tropical diseases and combat hepatitis, waterborne diseases, and other communicable diseases.
Target 3.8. Achieve universal health coverage, including financial risk protection, access to quality essential health-care services, and access to safe, effective, quality, and affordable essential medicines and vaccines for all.
Target 6.1. By 2030, achieve universal and equitable access to safe and affordable drinking water for all.
Target 6.2. By 2030, achieve access to adequate and equitable sanitation and hygiene for all and end open defecation, paying special attention to the needs of women and girls and those in vulnerable situations.
Target 13.3. Improve education, awareness-raising, and human and institutional capacity on climate change mitigation, adaptation, impact reduction, and early warning.

On the one hand, one could argue that the breadth of issues encapsulated in these tables serves to highlight the underpinning importance of NTDs for the attainment of a wider range of SDGs once and for all. It also supports a value for money argument by creating the logic that investment in NTDs would maximise returns across a broad range of SDGs and targets. Indeed, Dirk Engels made this point in a recent letter to *The Lancet*.

I do not share the opinion that the main outcome for the inclusion of the NTDs within the Sustainable Development Goals would be more money for the NTDs. Inclusion of NTD indicators and tracers will, on the contrary, help to maximise returns on investments in a broad portfolio of Sustainable Development Goal targets. (p 224) [8]

On the other hand, one could feasibly problematise the manner in which these connections have proliferated over time—being added to by different parties and via different outlets—insofar as they converge to produce a somewhat confusing and therefore harder to sell advocacy message for the NTDs. If we were to take anything away from the Big Three’s (HIV/AIDS, malaria and tuberculosis) experience of the MDGs, it is that *vertical* works in terms of lobbying, leveraging funds and even creating impact (even if everything else in the health sector is likely starved of oxygen while the gaze falls so resolutely on a single disease). Already, given the mainstream NTD rubric comprises 17 disparate diseases, NTDs are at a disadvantage when it comes to creating a clear message. To then try to sell the positive knock-on effects of dealing with small subsections of the larger disease umbrella to particular SDG targets, one can easily begin to become unstuck (so, for example, using NTDs as tracers for climate change could work so long as you’re only talking about the diseases with an amenable disease vector). The swelling ranks of the NTD lobby have likely been a contributing factor, with different players adding to the list of plausible NTD/SDG connections in recent years. We are not suggesting that this is a bad thing, nor are we calling into question any of the arguments that have been made in relation to the crosscutting nature of the NTDs; we are merely pointing out that advocacy tends to work best when the message is simple and coalesced around one voice. Hence, Dirk Engels’ letter to *The Lancet* in 2016 might well be construed as a calculated effort to refocus the NTD lobby around the two key issues for which NTDs could still be successful in winning tracer indicators: UHC and WASH.

Yet, even with this reduction in the agenda, more advocacy will be required to engage disease-endemic countries and convince them of these connections and the many knock-on advantages of tackling NTDs for human development. The 2014 update on the London Declaration reported that 74 countries—roughly two-thirds of all NTD-endemic countries—have national plans for NTD control. The report also highlighted that at the end of April 2013, African leaders took a major step towards promoting country ownership of NTD plans for the first time when they acknowledged the need to increase support for NTD programmes during the Sixth Conference of African Union Ministers of Health in Addis Ababa [14]. Yet, there is still a long way to go to foster genuine ownership of NTD programmes, as illustrated by the low uptake of donated drugs by endemic countries and by the reluctance of countries to co-invest in drug distribution. During an expert panel convened to celebrate the launch of the Countdown 2020 project in 2015, Professor David Molyneux suggested that it needn’t be that expensive for endemic countries to support the distribution of donated drugs—the unit cost of delivery would be US$0.25 per person per year, which is ~1% of the annual health budget of most African governments—therefore, in his opinion, it was a lack of political will barring the way (https://countdownonntds.wordpress.com/tag/fenwick/). Given the post-2015 agenda talks about moving “beyond an aid agenda” (p iii) [5], stimulating co-investment from national governments to implement NTD programmes will now become vital.

## Lobbying for an Indicator

The Inter-agency and Expert Group on Sustainable Development Goal Indicators (IAEG-SDGs) has been charged with arriving at a global indicator framework to monitor progress toward the newly adopted agenda. Therefore, and reflecting a current trend in development that that which is measured will be done (and by default or extension funded) the most recent pressing concern of the NTD lobby has been to identify and secure an indicator to help NTDs gain policy traction in the new agenda.

At the second meeting of the IAEG-SDGs in October 2015, NTDs received a tentative indicator under SDG 3.3 [15]. Watching the meeting, the technical and frankly tedious nature of proceedings could have obscured the fact that agreeing on indicators is an inherently political process. The SDGs will determine the global health budget for the next 15 years and, given the underfunding of the WHO NTD Roadmap [2], it has been vitally important for NTDs to gain a foothold in this process. Current funding commitments for NTDs (from various sources including Official Development Assistance, philanthropic giving, and national budgets) for the period of 2015–2020 have been projected at less than US$200 million a year; yet, the WHO has suggested that the total investment needed to support the NTD Roadmap for the 2015–2020 period is US$18 billion (p 24) [9]. This is a huge and highly problematic disparity.

The indicator that most concerns NTDs seeks to measure the “number of people requiring interventions against neglected tropical diseases” (p 9) [15]. This indicator works on the premise that disease-specific data already being collected to measure progress towards the *NTD Roadmap* can be synthesised to inform a single umbrella NTD indicator. The requisite data could thus be gathered from country systems and reported through joint requests and reporting forms for donated medicines and the integrated NTD database [16]. Routinely collecting and synthesising metadata on this scale remains a huge task, but one that has ultimately helped confirm the SDGs’ equity credentials by saying that no matter how mammoth the task (of intervening and of measuring), no longer can the world’s poorest people be allowed to fall through the cracks by literally not being counted. An indicator in this instance would create an imperative to strengthen and in some cases build from scratch systems that could genuinely improve the health of neglected populations.

In 2016, the WHO’s Dirk Engels impressed again the importance of using NTDs as “tracers of equity” relevant to the broader goals in his open letter in *The Lancet*. Here he made the point that while there is much to celebrate in the named inclusion of NTDs in target 3.3, “the inclusion of NTDs is no guarantee of success. The NTD community needs to continue to think outside of the box—the box numbered 3.3” (p 223) [8]. However, the aspiration that NTDs could additionally become tracers for the broader SDG framework is still to be decided.

The third meeting of the IAEG-SDGs took place 30 March–1 April 2016 and marked the beginning of the implementation process of the global SDG indicators. That meeting followed on closely from the 47th session of the United Nations Statistical Commission, which met 8–11 March 2016 and agreed to adopt the global indicator framework, concluding that the current proposal of 230 global indicators for the SDGs represented “a practical, good starting point" (http://www.un.org/sustainabledevelopment/blog/2016/03/un-statistical-commission-endorses-global-indicator-framework/). The third meeting of the IAEG-SDGs addressed the classification of indicators according to data availability and existing methodologies in order to identify statistical capacity building needs over the next 15 years. The IAEG-SDG also discussed mechanisms for future methodological reviews of global SDG indicators to allow transmission of data on SDG indicators from the national to the global level.

While the basic package of interventions to be included under target 3.8 on UHC remains to be defined (i.e., a package of essential interventions for which coverage can be measured), it is encouraging to note that in a preliminary document tabled for discussion at the recent IAEG-SDG meeting, a comment attached to the NTD indicator SDG 3.3.5 (*Number of people requiring interventions against Neglected Tropical Diseases*) suggests that certain NTD interventions are still in the running to be adopted as tracers.

Interventions [for indicator 3.3.5] are defined as preventive, curative, surgical, or rehabilitative treatment. Other (non-treatment) interventions (e.g., disease surveillance, morbidity management, and disability prevention, vector control, veterinary public health interventions) are to be addressed in the context of targets and indicators for Universal Health Coverage (UHC). (p 9) [17]

As Fitzpatrick and Engels remark in their recent paper, “target 3.8 would be the best place to deal with longer-term and more systemic interventions, including those dealing with surveillance and disability and inclusion” (p i17) [16], so it would make sense for these types of NTD interventions to find traction there.

## Conclusion: From Invisibility to Ubiquity

We are now firmly in the post-MDG era, but are still feeling our way into the Brave New World of the SDGs. The NTD lobby has been extraordinarily effective in building momentum and ultimately achieving recognition for NTDs within the new SDGs. This success is somewhat tempered by the sheer array of new goals, related targets, and uncertainty about how resources and commitments will map onto them.

The fight now is for traction within the emerging SDG Framework, and this requires a different focus. There is a need to shift from the limited number and international perspective of the MDGs to the much larger number of goals that need to be taken up and acted upon by a huge number of national governments. There is an opportunity here for NTDs to be leveraged throughout the SDGs; focusing on NTDs can assist nation states in grappling with the large array of new goals and targets. National governments must be—and can be—convinced of the crosscutting nature of NTD programmes and the benefits of mainstreaming NTD interventions, securing indicators and, thus, funding. There is a lot of hard work ahead, however.

There is a certain irony here that the previously “invisible” NTDs have gained prominence through their ubiquity within the SDGs, and this prominence is due in no small measure to the work of the NTD lobby thus far. Within the narrower rubric of the MDGs, the lower profile of NTDs was somewhat obscured until concerted efforts were made to underline how NTDs underpinned and interacted with the other goals and the very fabric of poverty itself. There is great value in NTDs being named in target 3.3, but there is still a challenge regarding relevance given the large number of other goals and targets, which may slice funding commitments rather more thinly than was the case with the MDGs. However, the ubiquity of NTDs in relation to the broader SDG agenda can come to the fore in relation to a greater number of goals and targets, especially those for which strong arguments can be made that NTDs may severely hamper progress: for example, goal 1 (end poverty) or goal 2 (end hunger), or where focusing on NTDs can drive progress towards specific targets, for example, 6.1 (achieve universal and equitable access to safe drinking water), 6.2 (achieve access to adequate and equitable sanitation and hygiene for all), and 3.8 (achieve UHC). From this perspective, an investment in NTDs becomes an investment in the broader sustainable development agenda [8,16].

Underlying and implicit in this is the ultimate aim of UHC. Here NTDs can act as both a focal point and a tracer indicator. Perhaps the newfound prominence and enduring ubiquity of NTDs is the mechanism to raise the prominence of the need for ubiquitous health coverage. If NTDs can become a mechanism to drive UHC, there may well be profound implications for the direction the NTD community choose to take next in their advocacy and action. There are a great many potential synergies to be built on, but also a great amount of coordination to be undertaken. Moreover, there is a risk to be managed as the NTD lobby looks to reconcile the WHO’s 2020 goals for the *NTD Roadmap* with the 2030 timeframe of the SDGs [18].
